# Anemia and Iron Deficiency in Children with Chronic Kidney Disease (CKD): Data from the Know-Ped CKD Study

**DOI:** 10.3390/jcm8020152

**Published:** 2019-01-29

**Authors:** Keum Hwa Lee, Eujin Park, Hyun Jin Choi, Hee Gyung Kang, Il-Soo Ha, Hae Il Cheong, Young Seo Park, Heeyeon Cho, Kyoung Hee Han, Seong Heon Kim, Min Hyun Cho, Joo Hoon Lee, Jae Il Shin

**Affiliations:** 1 Department of Pediatrics, Yonsei University College of Medicine, Yonsei-ro 50, Seodaemun-gu, C.P.O. Box 8044, Seoul 03722, Korea; azsagm@yuhs.ac; 2 Division of Pediatric Nephrology, Severance Children’s Hospital, Seoul 03722, Korea; 3 Department of Pediatrics, Hallym University Kangnam Sacred Heart Hospital, Seoul 07441, Korea; eujinpark@hallym.or.kr; 4 Department of Pediatrics, Seoul National University Children’s Hospital & Seoul National University College of Medicine, Seoul 03080, Korea; h_choi@daum.net (H.J.C.); kanghg@snu.ac.kr (H.G.K.); ilsooha@snu.ac.kr (I.-S.H.); cheonghi@snu.ac.kr (H.I.C.); 5 Department of Pediatrics, Asan Medical Center Children’s Hospital, University of Ulsan College of Medicine, 88, Olympic-ro 43-Gil, Songpa-Gu, Seoul 05505, Korea; yspark@amc.seoul.kr; 6 Department of Pediatrics, Samsung Medical Center, Sungkyunkwan University School of Medicine, Seoul 06351, Korea; heeyeon1.cho@samsung.com; 7 Department of Pediatrics, Jeju National University School of Medicine, Jeju 63241, Korea; hansyang78@gmail.com; 8 Department of Pediatrics, Pusan National University Children’s Hospital, Yangsan 50612, Korea; pedkimsh@pusan.ac.kr; 9 Department of Pediatrics, Kyungpook National University, School of Medicine, Daegu 41404, Korea; chomh@knu.ac.kr; 10 Institute of Kidney Disease Research, Yonsei University College of Medicine, Seoul 03722, Korea

**Keywords:** anemia, iron deficiency, chronic kidney disease

## Abstract

Children with chronic kidney disease (CKD) are at high risk of anemia, an important risk factor for cardiovascular disease and poor quality of life. The present study used baseline data from the Korean cohort study for Outcome in patients With Pediatric Chronic Kidney Disease (KNOW-PedCKD). A Total of 437 patients was included in the analyses excluding missing data. The characteristics of patients with and without anemia and those of patients with and without iron deficiency were compared. Logistic regression analysis and Pearson correlation were conducted to evaluate associated risk factors and correlations in children with CKD. Anemia in children with CKD was associated with older age, low body weight and body mass index (BMI) z-score, birth age, preceding glomerulonephritis, decreased estimated glomerular filtration rate (eGFR), low levels of serum albumin and calcium, high levels of serum intact parathyroid hormone (iPTH), and serum phosphorus. Anemia was correlated positively with changes in the BMI z-score, body weight, and serum albumin and cholesterol levels, but correlated negatively with serum calcium, iPTH, ferritin levels, and transferrin saturation. Iron deficiency in children with CKD was associated with young age, low hemoglobin and serum ferritin levels, high BMI z-scores, and low levels of serum iPTH. This is the first nationwide cohort study of anemia in Korean children with CKD and the first prospective pediatric CKD cohort study in Asia. The study results demonstrated that anemia and iron deficiency are affected by various factors, including age, BMI, and levels of serum iPTH. To improve the retrospective outcome of affected children, it is important to understand the effect of each of these factors and to attempt an early intervention to prevent anemia and iron deficiency by regular measurement of these parameters in children at risk.

## 1. Introduction

Anemia is a major complication in children with chronic kidney disease (CKD). Data from the North American Pediatric Renal Trials and Collaborative Studies (NAPRTCS) revealed the prevalence of anemia in children with CKD, ranging from 73 to 93%, depending on the CKD stage [1]. Among children with CKD, anemia is an important risk factor for the development and progression of cardiovascular disease, including left ventricular hypertrophy [2,3]. In addition, anemia negatively affects the quality of life of patients and their caregivers [4,5].

The chronic inflammatory state in patients with CKD results in decreased erythropoiesis in the bone marrow, reduced production of erythropoietin (EPO) in the kidneys, and impaired iron absorption and mobilization due to increased production of hepcidin in the liver. Uremia, oxidative stress, and nutritional deficiencies also contribute to anemia in CKD [6]. Therefore, although therapeutic targets involving hemoglobin and iron levels are controversial, use of an erythropoiesis-stimulating agent (ESA) and iron supplementation are key approaches to the management of anemia in CKD patients [7,8]. However, treatment of anemia in children with CKD is known to be less effective than that in adults [9], and studies of anemia and iron deficiency are limited due to the small numbers of pediatric patients with CKD.

The objectives of this study were to examine the characteristics of patients with anemia and iron deficiency among children with CKD and to identify associated risk factors and correlations using baseline data from the Korean cohort study for Outcome in patients With Pediatric Chronic Kidney Disease (KNOW-PedCKD).

## 2. Materials and Methods

### 2.1. Study Design and Population

The KNOW-PedCKD study is a nationwide observational prospective cohort study of pediatric CKD patients with CKD stages 1–5 recruited from seven major pediatric nephrology centers in Korea. Details on the study design and objectives have been published previously [10]. For the current analysis, we included subjects enrolled from April 2011 to February 2016 (*n* = 437). A total of 437 patients was included in the analysis. If the individual tests have missing data, they were excluded in the final analysis. 

### 2.2. Clinical and Laboratory Measurements

CKD was defined and staged according to the Kidney Disease Improving Global Outcomes (KDIGO) criteria [7]. For patients enrolled in the KNOW-PedCKD study, the estimated glomerular filtration rate (eGFR) was calculated using the bedside Chronic Kidney Disease in Children (CKiD) formula [11]. Data on medical history and medication use were collected through a self-administered questionnaire. Anemia was defined as hemoglobin below the 5th percentile for age/sex or treatment with an erythropoiesis-stimulating agent (ESA) [7,12]. Iron deficiency was defined as serum ferritin <100 ng/mL and transferrin saturation <20% [7,8]. Hypertension was defined as the average value of the systolic and/or diastolic BP measurements above the 95th percentile for age, sex, and height. The presence of comorbidity included heart failure, arrhythmia, urinary tract infection, diabetes, retinopathy, sensorineural hearing loss, developmental delay, and metabolic syndrome.

### 2.3. Statistical Analyses

Categorical variables expressed as percentages were compared between groups with the Chi-square test and Fisher’s exact test. Continuous variables were expressed as a mean ± standard deviation and analyzed by the Kruskal–Wallis test. A logistic regression analysis was conducted to determine the odds ratios (ORs) and confidence intervals (CIs) of associated risk factors for anemia and iron deficiency in the cohort. The correlations among characteristics of anemia in the cohort were analyzed with a Pearson *r* correlation. All statistical analyses were performed using SAS statistical software (SAS system for Windows, version 9.4; SAS institute, Cary, NC, USA).

## 3. Results

### 3.1. Baseline Characteristics of Overall CKD Cohort

The demographic characteristics and clinical manifestations of patients with CKD are shown in Table 1. The mean age of the patients was 9.5 ± 5.3 years, and most patients were found to be in the middle 10s (43.9%). Among the CKD patients, male patients were more frequent than female patients regardless of age (*n* = 299 versus *n* = 138). The most common disease leading to CKD was renal hypoplasia (41.4%), while 66 patients had kidney dysplasia with/out vesico-ureteral reflux (VUR) (15.1%), and 27.7% had glomerulonephritis. Overall, 27.2% of patients were in CKD stage 2, while a minority (3.9%) had CKD stage 5 in a pre-dialysis state.

### 3.2. CKD Patients with Anemia

Characteristics of the CKD patients with and without anemia are listed in Table 2. Overall, older age (*p* < 0.001), glomerulonephritis as a preceding disease (*p* < 0.001), higher CKD stages (*p* < 0.001), higher diastolic blood pressure (DBP) (*p* = 0.029), lower body weight (*p* = 0.036), proteinuria (*p* < 0.001), and use of a renin-angiotensin system inhibitor (*p* = 0.002) were significantly associated with CKD patients with anemia, compared to those without. CKD patients with anemia were treated with erythropoietin (EPO)-stimulating agents (21.6%, *p* = 0.001) and supplemental iron therapy (36.3%, *p* = 0.001), and showed significantly lower ferritin levels (*p* < 0.001) than those without anemia. There were no significant differences in sex distribution, platelet counts, systolic blood pressure, or transferrin saturation according to the presence of anemia.

Patients with CKD showed a bimodal distribution with low hemoglobin levels at ages 0–2 years (11.14 ± 1.64 g/dL) and at ages 6–12 years (11.65 ± 1.79 g/dL) (Table 3). Hemoglobin levels decreased significantly with increases in CKD stage, with a mean of 12.22 ± 1.94 g/dL (*p* < 0.001) (Table 4). Hemoglobin distribution according to the disease preceding CKD is summarized in Table 5. Patients with primary glomerulonephritis had significantly lower hemoglobin levels, compared to other kidney diseases (*p* = 0.003).

In children with CKD who did not receive ESA, anemia was associated with older age (OR 1.075, *p* < 0.001), low weight (OR 0.853, *p* = 0.008), low body mass index (BMI) z-score (OR 0.779, *p* = 0.003), existence of glomerulonephritis (OR 3.263, *p* < 0.001), high levels of ionized parathyroid hormone (iPTH) (OR 1.007, *p* < 0.001) and serum phosphorus (OR 1.773, *p* < 0.001), low serum albumin (OR 0.498, *p* < 0.001) and calcium (OR 0.494, *p* < 0.001) levels, and low eGFR (OR 0.966, *p* < 0.001) in the univariate logistic regression. The multiple logistic regression analysis revealed the existence of glomerulonephritis (OR 3.263, *p* < 0.001) to be an independent risk factor associated with anemia in children with CKD (Table 6).

Anemia correlated positively with changes in BMI z-score (*r* = 0.434, *p* < 0.001), weight (*r =* 0.324, *p* < 0.001), serum albumin (*r* = 0.288, *p* < 0.001), and cholesterol (*r* = 0.398, *p* < 0.001), but correlated negatively with serum calcium (*r* = −0.403, *p* < 0.001), iPTH (*r* = −0.616, *p* < 0.001), ferritin (*r* = −0.119, *p* = 0.019), and transferrin saturation (*r* = −0.253, *p* < 0.001) (Table 7).

### 3.3. CKD Patients with Iron Deficiency

Characteristics of the CKD patients with iron deficiency are summarized in Table 8. Age (*p* = 0.002), existence of glomerulonephritis as a preceding disease (*p* = 0.017), systolic blood pressure (*p* = 0.036), and presence of comorbidities (*p* = 0.009) were significantly higher in patients with iron deficiency than in those without. Other variables, including sex, weight, CKD stages, and use of iron supplementation agents, did not differ between the two groups.

In CKD patients with iron deficiency, ferritin levels were significantly lower in the 2–6 year-old age group (44.86 ± 53.08 ng/mL, *p* < 0.001); however, the lowest age group with transferrin saturation was 0–2 years (21.68 ± 13.51%, *p* = 0.001) (Table 3). Interestingly, ferritin levels showed a significant increase as the CKD stage increased (*p* < 0.001). In contrast, there were no significant differences in transferrin saturation across the CKD stages (Table 4). As shown in Table 5, kidney dysplasia with/out VUR, pyelonephritis, and children with interstitial nephritis had the lowest ferritin levels, compared to other kidney diseases (55.79 ± 43.59 ng/mL, *p* < 0.001). Transferrin saturation showed a significantly lower percentage in the congenital, hereditary, cystic disease group (24.28 ± 11.44%, *p* < 0.001).

In children with CKD who did not take iron supplements, iron deficiency was associated with younger age (OR 0.908, *p* < 0.001), lower hemoglobin (OR 0.843, *p* = 0.015), and lower ferritin (OR 0.980, *p* < 0.001) levels in the univariate logistic regression analysis. The multiple logistic regression analysis demonstrated that an increased BMI z-score (OR 1.318, *p* = 0.024) and decreased iPTH (OR 0.993, *p* = 0.007), hemoglobin (OR 0.693, *p* < 0.001), and ferritin (OR 0.976, *p* < 0.001) levels were risk factors associated with iron deficiency in children with CKD (Table 9).

## 4. Discussion

Anemia and iron deficiency are important complications in pediatric patients with CKD. The aim of aggressive treatment of anemia is to avoid regular red blood cell transfusions and to improve the quality of life, cognitive function, exercise capacity, and cardiovascular function of children [13]. The best treatment options for anemia and iron deficiency are EPO-stimulating and iron supplementation agents; nonetheless, their use in hemodialysis or peritoneal dialysis patients is known to be less effective than that in adults [14]. Therefore, it is important to identify the status of anemia and iron deficiency and to actively correct these in pediatric CKD patients.

KNOW-PedCKD is the first prospective pediatric CKD cohort study about the clinical characteristics and prevalence of anemia and iron deficiency in Asia. In addition, this is the first attempt to analyze risk factors for anemia in CKD children. Other pediatric CKD cohort studies, such as CKiD or “The Functional Outcomes in Adolescent CKD study”, showed hemoglobin levels and only in part reported the prevalence of anemia [15,16] In contrast, we performed comprehensive analyses firstly in the pediatric area.

In our KNOW-PedCKD cohort study, the proportion of CKD patients with anemia was the lowest at 2–5 years of age and then increased with older age. This result was comparable to that reported in a previous study on a pediatric CKD cohort, NAPRTCS [1]. Thus, school-aged patients of ages 12–17 years with CKD are more likely to have anemia, making it is necessary for clinicians to detect and treat anemia appropriately.

Individual studies, such as the ItalKid study [17], the British Association of Pediatric Nephrology [18], and NAPRTCS [1,19], have reported the major causes of CKD. These studies suggest that glomerulonephritis is the cause of end-stage renal disease (ESRD) in 20% of cases, while congenital abnormalities of the kidney and urinary tract (CAKUT) occur in 50% of patients. Other congenital diseases (CG) and hereditary diseases (HD) are considered causative in 20% of patients [1,17,18,19].

However, studies are lacking with regard to the relationships between diseases leading to CKD and anemia. Interestingly, our study showed that anemia is more severe in patients with glomerulonephritis than in those without. Recent evidence indicates that medications for the blockade of the renin-angiotensin system (RAS) pathway to reduce proteinuria in glomerulonephritis modulate erythropoiesis by regulation of angiotensin II signaling. Angiotensin II is involved in the proliferation and differentiation of hematopoietic cell types, especially those of red blood cell lineage. Both angiotensin-converting enzyme (ACE) inhibitors and angiotensin receptor blockers (ARB) induce adverse effects related to decreased hematocrits [20]. Notwithstanding, iron deficiency was more prominent in patients with non-glomerulonephritis (kidney dysplasia with/out VUR, pyelonephritis, interstitial nephritis, congenital disease, hereditary disease, cystic disease, and others) (Table 8).

Ferritin increased as the CKD stage increased in our cohort. According to another report, ferritin itself could act as an inflammatory marker [21]. In addition, according to Goyal et al., cytokines, such as hepcidin and high-sensitivity C-reactive protein (hs-CRP), increase with increasing CKD stages and ferritin showed a positive correlation with hepcidin [22]. This indicates that ferritin itself can reflect inflammation in CKD patients. Because of the limited possibility to take blood in the pediatric population, the fact that ferritin alone can detect both inflammation and iron deficiency in pediatric CKD patients is a very important achievement in our study (Table 4).

The correlation results in our study indicated that anemia was positively correlated with low body weight and BMI z-score (*r =* 0.324, *p* < 0.001 and *r* = 0.434, *p* < 0.001, respectively, Table 7). Although it was not found to be an independent risk factor in the multivariate analysis, anemia and growth are closely related in children with CKD. The underlying mechanism to explain low body weight and BMI z-score with anemia are not very well-understood, although both may be related to CKD progression. Low body weight and BMI z-score are considered to be results of the progression of CKD, a combination of anorexia, increased energy expenditure, and muscle wasting [23], and anemia has also been proposed to be associated with renal function itself. The relationship between eGFR and anemia in childhood CKD has been studied in several studies [13,23]. Recently, Atkinson et al. analyzed data calculated using the CKiD and reported that, when the eGFR is reduced by 20%, the hemoglobin level tends to decrease by 0.2–0.4 g/dL [15]. Similar to the CKiD results, the distribution of anemia according to CKD stage in the KNOW-PedCKD cohort also increased according to higher CKD stages (Table 2).

Hypertension is one of the major causes of CKD worldwide [24]. Our results indicated that hypertension is associated with anemia based on diastolic blood pressure and iron deficiency based on systolic blood pressure in children with CKD (Table 2 and Table 8). However, in the univariate and multivariate analyses, hypertension was not independently associated with anemia and iron deficiency (Table 6 and Table 9).

Anemia and iron deficiency are associated risk factors of CKD. Despite the established treatment of anemia and iron deficiency in CKD patients, our result suggests the importance of simultaneous use of EPO-stimulating agents and iron supplements as an effective treatment approach.

There are several limitations in this study. First, there could be unavailable data or information of patients of the CKD cohort. Second, we could not include the patients’ diets and calorie intake in this report. Third, we may not have excluded potential significant inter-study variations in the study setting. In addition, it is not clear in CKD patients as to whether these indicators are the effect of CKD itself or impact of anemia. We plan in future studies to examine the relationship between the impact of anemia on quality of life, cognitive function, exercise capacity, and cardiovascular function and anemia in the KNOW-pedCKD cohort.

## 5. Conclusions

This article is noteworthy because it is the first multicenter cohort study investigating the impact of anemia in Korean pediatric patients with CKD and the first prospective pediatric CKD cohort study in Asia. Supported by clinical evidence, our results indicate that anemia and iron deficiency are key factors associated with CKD in pediatric patients. Understanding the multiple risk factors causing anemia in CKD will improve the outcomes of children with CKD. Furthermore, ESA and iron-replacement therapy have revolutionized the treatment of anemia in children and the negative effects of anemia can be improved by these treatments. Additional studies are necessary to address the issue of anemia in pediatric CKD according to each patient’s diet or quality of life in the future.

## Figures and Tables

**Table 1:** Baseline demographics of the chronic kidney disease (CKD) patient cohort.

Variable	Total Patients (%)
	(*n* = 437)
**Age (years)**	9.5 ± 5.3
0–2 years	43 (9.8)
2–6 years	77 (17.6)
6–12 years	125 (28.6)
12–17 years	192 (43.9)
**Sex (male/female)**	299/138
**Disease preceding CKD**	
Primary glomerulopathy *	61 (14.0)
Secondary glomerulopathy *	60 (13.7)
Kidney dysplasia with/out vesico-ureteral reflux	66 (15.1)
Hypoplasia	181 (41.4)
Other	69 (15.8)
**CKD stages**	
I	79 (18.1)
II	119 (27.2)
IIIa ^※^	74 (16.9)
IIIb ^※^	71 (16.3)
IV	77 (17.6)
V	17 (3.9)
**Birth weight (kg)**	3.0 ± 0.7
**Birth age (weeks)**	38.3 ± 3.1
**Disease duration (years) †**	5.7 ± 4.7
**Duration of aggravated renal function (years) ^‡^**	2.9 ± 3.4

Numerical values are reported as *n* (%); CKD, chronic kidney disease; n, number of patients. * More detailed data are described in supplementary Table S1; ^※^ CKD stage III is divided into subgroup stage IIIa and IIIb according to the eGFR (estimated glomerular filtration rate), stage IIIa is an eGFR between 45 and 59 and Stage 3b is an eGFR between 30 and 44; † Disease duration refers to the years between the date of the baseline data collection and the CKD diagnosis date; ^‡^ Duration of aggravated renal function means the years between the date of the baseline data collection and the date of onset of the preceding disease to CKD.

**Table 2:** Comparisons of characteristics of patients with anemia in the CKD cohort analysis.

Variable	Patients with Anemia (*n* = 172)	Patients without Anemia (*n* = 261)	*P* Value
**Age (years)**	172	261	<0.001 ††
0–2 years	11 (6.4)	28 (10.7)	
2–6 years	17 (9.9)	60 (23.0)	
6–12 years	49 (28.5)	76 (29.1)	
12–17 years	95 (55.2)	97 (37.2)	
**Sex (male/female)**	119/53	175/86	0.641 ††
**Disease preceding CKD**	150	228	<0.001 ††
Glomerulonephritis	49 (32.7)	30 (13.2)	
Non-glomerulonephritis	101 (67.3)	198 (86.8)	
**CKD stages**	172	261	<0.001 ††
I	8 (4.7)	71 (27.2)	
II	26 (15.1)	92 (35.2)	
IIIa ^※^	32 (18.6)	41 (15.7)	
IIIb ^※^	38 (22.1)	33 (12.6)	
IV	54 (31.4)	21 (8.0)	
V	14 (9.9)	3 (1.1)	
**Weight (kg)**	171	255	0.036 ††
Low weight	60 (35.1)	61 (23.9)	
Normal	107 (62.6)	184 (72.2)	
Overweight	4 (2.3)	10 (3.9)	
**Height (cm)**	171	255	0.058 †
Short stature	48 (28.1)	49 (19.2)	
Normal	122 (71.4)	201 (78.8)	
Tall stature	1 (0.6)	5 (2.0)	
**Hypertension**	156	216	
SBP hypertension	24 (15.4)	34 (15.7)	0.926 ††
DBP hypertension	43 (27.6)	39 (18.1)	0.029 ††
**Proteinuria (mg/mg)**	172	261	<0.001 ††
<0.5	69 (15.9)	162 (37.4)	
≥0.5	103 (37.4)	99 (22.9)	
**Ferritin (ng/mL)**	163	233	<0.001 ††
≥100	51 (31.3)	33 (14.2)	
<100	112 (68.7)	200 (85.8)	
**Transferrin saturation (%)**	170	253	0.694 ††
<20	54 (31.8)	85 (33.6)	
≥20	116 (68.2)	168 (66.4)	
**Use of EPO stimulating agents**	167	261	0.001 ††
Yes *	36 (21.6)	7 (2.7)	
**Iron supplementation treatment**	171	261	0.001 ††
Yes **	62 (36.3)	29 (11.1)	
**Use of renin-angiotensin system (RAS) inhibitors**	172	261	0.002 ††
Yes ***	110 (70.0)	123 (47.1)	

Numerical values are reported as *n* (%); CKD, chronic kidney disease; *n*, number of patients; EPO, erythropoietin; SBP, systolic blood pressure; DBP, diastolic blood pressure; ^※^ CKD stage III is divided into subgroup stage IIIa and IIIb according to the eGFR (estimated glomerular filtration rate), stage IIIa is an eGFR between 45 and 59 and Stage 3b is an eGFR between 30 and 44; * EPO stimulating agents: Epoetin alfa, Epoetin beta; ** Iron agents: any agent including intravenous or oral intake; *** RAS inhibitors: Angiotensin-converting enzyme inhibitors, angiotensin II receptor blockers; † Statistically significant differences were demonstrated using Fisher’s exact test; †† Statistically significant differences were demonstrated using the Chi-square test.

**Table 3:** Hemoglobin, transferrin saturation, and ferritin levels stratified by age in the CKD cohort.

	Total	0–2 Years	2–6 Years	6–12 Years	12–18 Years	*P* Value
	(*n* = 437)	(*n* = 43)	(*n* = 77)	(*n* = 125)	(*n* = 192)	
**Hemoglobin (g/dL)**	12.15 ± 1.94	11.14 ± 1.64	12.34 ± 1.43	11.65 ± 1.79	12.55 ± 2.12	<0.001 *
Male	12.37 ± 2.01	11.35 ± 1.92	12.58 ± 1.34	11.6 ± 1.8	13.04 ± 2.11	<0.001 *
Female	11.9 ± 1.74	11.43 ± 1.7	12.39 ± 1.48	11.85 ± 1.71	11.8 ± 1.87	0.227 *
**Transferrin saturation (%)**	27.86 ± 16.49	21.68 ± 13.51	23.05 ± 10.87	29.84 ± 21.63	29.25 ± 14.15	0.001 *
Male	27.47 ± 16.02	21.35 ± 10.89	23.38 ± 11.51	29.19 ± 23.02	29.02 ± 11.53	0.001 *
Female	27.68 ± 15.92	20.66 ± 15.99	22.9 ± 9.96	30.23 ± 13.92	30.31 ± 18.53	0.04 *
**Ferritin (ng/mL)**	99.47 ± 211.51	96.2 ± 114.5	44.86 ± 53.08	111.03 ± 270.89	113.12 ± 217.17	<0.001 *
Male	93.43 ± 172.19	105.65 ± 129.99	49.6 ± 65.59	105.02 ± 262.15	97.21 ± 114.86	<0.001 *
Female	112.75 ± 279.6	76.44 ± 73.84	38.1 ± 26.41	128.25 ± 298.59	148.9 ± 351.2	0.016 *

CKD, chronic kidney disease; *n*, number of patients; * Statistical significance was demonstrated using the Kruskal–Wallis test.

**Table 4:** Hemoglobin, transferrin saturation, and ferritin levels stratified by CKD stage.

	Total (*n* = 437)	I (*n* = 79)	II (*n* = 119)	IIIa ^※^ (*n* = 74)	IIIb ^※^ (*n* = 71)	IV (*n* = 77)	V (*n* = 17)	*P* Value
**Hemoglobin (g/dL)**	12.22 ± 1.94	13.1 ± 1.46	13.03 ± 1.73	12.34 ± 1.75	11.75 ± 1.77	10.85 ± 1.72	10.14 ± 2.17	<0.001 *
Male	12.37 ± 2.01	13.41 ± 1.49	13.21 ± 1.83	12.48 ± 1.67	11.84 ± 1.82	10.84 ± 1.8	10.38 ± 2.18	<0.001 *
Female	11.9 ± 1.74	12.39 ± 1.14	12.65 ± 1.46	12.05 ± 1.93	11.58 ± 1.69	10.87 ± 1.56	9.58 ± 2.31	<0.001 *
**Transferrin saturation (%)**	27.54 ± 15.97	27.87 ± 23.68	26.06 ± 12.56	26.88 ± 14.12	29.34 ± 13.66	27.19 ± 13.29	33.19 ± 20.44	0.449 *
Male	27.47 ± 16.02	28.65 ± 26.55	27.43 ± 12.57	25.55 ± 12.13	28.32 ± 10.63	26.74 ± 13.34	30.32 ± 15.85	0.722 *
Female	27.68 ± 15.92	25.91 ± 14.51	23.03 ± 12.19	29.58 ± 17.48	31.23 ± 18.06	28.25 ± 13.41	40.1 ± 29.93	0.338 *
**Ferritin (ng/mL)**	99.47 ± 211.51	53.27 ± 51.2	81.58 ± 135.93	83.15 ± 131.44	122.96 ± 335.38	139.63 ± 284.45	201.81 ± 231.99	<0.001 *
Male	93.43 ± 172.19	60.25 ± 57.3	78.42 ± 75.42	77 ± 111.18	61.98 ± 59.64	171.92 ± 342.43	191.83 ± 263.38	<0.001 *
Female	112.75 ± 279.6	33.51 ± 16.37	88.6 ± 218.8	96.26 ± 168.89	239.62 ± 555.72	75.03 ± 60.18	225.78 ± 153.27	0.013 *

CKD, chronic kidney disease; *n*, number of patients; ^※^ CKD stage III is divided into subgroup stage IIIa and IIIb according to the eGFR (estimated glomerular filtration rate), stage IIIa is an eGFR between 45 and 59 and Stage 3b is an eGFR between 30 and 44; * Statistical significance was demonstrated using the Kruskal–Wallis test.

**Table 5:** Hemoglobin, transferrin saturation, and ferritin levels stratified by disease preceding CKD.

	Total (*n* = 437)	1’ GN (*n* = 61)	2’ GN (*n* = 60)	RN/PN/IN (*n* = 66)	CG/HD/CD (*n* = 181)	Other (*n* = 69)	*P* Value
**Hemoglobin (g/dL)**	12.22 ± 1.94	11.39 ± 1.94	12.3 ± 1.5	12.77 ± 1.87	12.29 ± 1.88	12.18 ± 2.29	0.003 *
Male	12.37 ± 2.01	11.64 ± 2.09	12.41 ± 1.65	12.94 ± 1.84	12.36 ± 1.92	12.34 ± 2.46	0.043 †
Female	11.9 ± 1.74	10.92 ± 1.55	12.14 ± 1.26	11.81 ± 1.83	12.15 ± 1.79	11.9 ± 1.97	0.097
**Transferrin saturation (%)**	27.54 ± 15.97	36.44 ± 27.36	27.66 ± 13.13	25.92 ± 10.6	24.28 ± 11.44	29.86 ± 16.6	<0.001 *
Male	27.47 ± 16.02	36.31 ± 30.69	27.47 ± 11.12	26.77 ± 10.45	24.55 ± 11.74	28.49 ± 14.1	0.009 *
Female	27.68 ± 15.92	36.69 ± 20.01	27.93 ± 15.93	21.25 ± 10.77	23.73 ± 10.85	32.54 ± 20.75	0.056
**Ferritin (ng/mL)**	99.47 ± 211.51	90.08 ± 77.22	92.98 ± 183.59	55.79 ± 43.59	70.03 ± 102.28	242.3 ± 460.64	<0.001 *
Male	93.43 ± 172.19	101.24 ± 87.55	70.11 ± 64.22	59.46 ± 45.55	76.02 ± 115.35	204.69 ± 387.22	<0.001 *
Female	112.75 ± 279.6	67.19 ± 43.32	127.8 ± 280.81	34.19 ± 19.77	57.59 ± 66.85	310.36 ± 574.81	0.003 *

CKD, chronic kidney disease; *n*, number of patients; GN, glomerulonephritis; RN, kidney dysplasia with/out vesico-ureteral reflux; PN, pyelonephritis, IN, interstitial nephritis; CG, congenital disease; HD, hereditary disease; CD, cystic disease; * Statistical significance was demonstrated using the Kruskal–Wallis test; † Statistical significance was demonstrated using the Anova test.

**Table 6:** Associated risk factors for anemia in patients with CKD who did not take erythropoietin-stimulating agents.

	Univariate Logistic Regression in Anemia Patients		Multivariable Logistic Regression in Anemia Patients	
	Unadjusted OR (95% CI)	*P*-Value	Adjusted OR (95% CI)	*P* Value
**Age**	1.075 (1.031, 1.121)	0.0006	-	-
**Weight z-score**	0.853 (0.758, 0.960)	0.0081	-	-
**Height z-score**	0.901(0.79, 1.029)	0.1247	-	-
**BMI z-score**	0.779 (0.661, 0.918)	0.0029	-	-
**Birth age**	1.132 (1.039, 1.233)	0.0046	-	-
**Glomerulonephritis**	3.263 (1.905, 5.590)	<0.0001	3.263 (1.905, 5.590)	<0.0001
**Hypertension**	1.184 (0.731, 1.915)	0.4928	-	-
**Serum albumin**	0.498 (0.355, 0.698)	<0.0001	-	-
**eGFR**	0.966 (0.957, 0.975)	<0.0001	-	-
**iPTH**	1.007 (1.004, 1.011)	<0.0001	-	-
**Serum Ca**	0.494 (0.389, 0.628)	<0.0001	-	-
**Serum P**	1.773 (1.352, 2.324)	<0.0001	-	-
**Urine protein creatinine ratio**	1.001 (0.968, 1.035)	0.9482	-	-
**Hemoglobin**	0.138 (0.091, 0.208)	<0.0001	-	-
**Ferritin**	1.001 (1.000, 1.003)	0.0667	-	-
**Transferrin saturation**	1.003 (0.990, 1.016)	0.6548	-	-
Iron supplementation use **	3.652 (2.058, 6.482)	<0.0001	-	-

CKD, chronic kidney disease; OR, odds ratio; CI, confidence interval; BMI, body mass index; GFR, estimated glomerular filtration rate; iPTH, ionized parathyroid hormone; Ca, serum calcium; P, serum phosphorus; ** Iron agents: any agents including intravenous or oral intake.

**Table 7:** Correlations among key characteristics of anemic children in the CKD cohort.

	Hb	Reti	Tran Sat	Ferritin	iPTH	eGFR	Alb	Ca	P	Chole	Weight	BMI−z
**Hb**	.	0.0282 0.5569	−0.2531 <0.0001 (−)	−0.119 0.0194 (−)	−0.6155 <0.0001 (−)	0.0297 0.5473	0.2877 <0.0001	−0.4025 <0.0001 (−)	0.0993 0.1048	0.3976 <0.0001	0.3235 <0.0001	0.4339 <0.0001
**Reti**	0.0282 0.5569	.	−0.1441 0.0026 (−)	0.1535 0.0013	−0.1766 0.0002 (−)	0.3302 <0.0001	0.1117 0.0244	−0.038 0.5237 (−)	0.1182 0.0537	−0.0391 0.4257 (−)	0.0082 0.8654	0.3593 <0.0001
**Tran sat**	−0.2531 <0.0001 (−)	−0.1441 0.0026 (−)	.	0.1941 <0.0001	0.2095 <0.0001	0.0062 0.8994	−0.0082 0.8688 (−)	0.2913 <0.0001	0.0791 0.1975	−0.0846 0.0842 (−)	−0.0724 0.1343 (−)	−0.3134 <0.0001 (−)
**Ferritin**	−0.119 0.0194 (−)	0.1535 0.0013	0.1941 <0.0001	.	0.1567 0.0011	0.2422 <0.0001	0.1265 0.0107	0.0746 0.2100	0.0901 0.1422	−0.1143 0.0194 (−)	−0.0186 0.7004 (−)	−0.0317 0.5087 (−)
**iPTH**	−0.6155 <0.0001 (−)	−0.1766 0.0002 (−)	0.2095 <0.0001	0.1567 0.0011	.	−0.1159 0.0185 (−)	−0.1963 <0.0001 (−)	0.2178 0.0002	−0.0552 0.3691 (−)	−0.2078 <0.0001 (−)	−0.0933 0.0548 (−)	−0.3635 <0.0001 (−)
**eGFR**	0.0297 0.5473	0.3302 <0.0001	0.0062 0.8994	0.2422 <0.0001	−0.1159 0.0185 (−)	.	0.1851 0.0002	−0.0656 0.2759 (−)	0.0993 0.1075	0.0253 0.6117	−0.1289 0.0092 (−)	0.0957 0.052
**Alb**	0.2877 <0.0001	0.1117 0.0244	−0.0082 0.8688 (−)	0.1265 0.0107	−0.1963 <0.0001 (−)	0.1851 0.0002	.	−0.123 0.0431 (−)	−0.028 0.6528 (−)	0.0632 0.2074	0.0154 0.7589	0.0693 0.1631
**Ca**	−0.4025 <0.0001 (−)	−0.038 0.5237 (−)	0.2913 <0.0001	0.0746 0.2100	0.2178 0.0002	−0.0656 0.2759 (−)	−0.123 0.0431 (−)	.	0.0391 0.587	−0.2098 0.0004 (−)	−0.0454 0.4455 (−)	−0.2708 <0.0001 (−)
**P**	0.0993 0.1048	0.1182 0.0537	0.0791 0.1975	0.0901 0.1422	−0.0552 0.3691 (−)	0.0993 0.1075	−0.028 0.6528 (−)	0.0391 0.587	.	0.0666 0.2821	0.415 <0.0001	0.1246 0.0415
**Chole**	0.3976 <0.0001	−0.0391 0.4257 (−)	−0.0846 0.0842 (−)	−0.1143 0.0194 (−)	−0.2078 <0.0001 (−)	0.0253 0.6117	0.0632 0.2074	−0.2098 0.0004 (−)	0.0666 0.2821	.	0.1959 <0.0001	0.2174 <0.0001
**Weight**	0.3235 <0.0001	0.0082 0.8654	−0.0724 0.1343 (−)	−0.0186 0.7004 (−)	−0.0933 0.0548 (−)	−0.1289 0.0092 (−)	0.0154 0.7589	−0.0454 0.4455 (−)	0.415 <0.0001	0.1959 <0.0001	.	0.214 <0.0001
**BMI−z**	0.4339 <0.0001	0.3593 <0.0001	−0.3134 <0.0001 (−)	−0.0317 0.5087 (−)	−0.3635 <0.0001 (−)	0.0957 0.052	0.0693 0.1631	−0.2708 <0.0001 (−)	0.1246 0.0415	0.2174 <0.0001	0.214 <0.0001	.

CKD, chronic kidney disease; Hb, hemoglobin; Reti, reticulocyte count; Tran sat, transferrin saturation; iPTH, ionized parathyroid hormone; eGFR, estimated glomerular filtration rate; Alb, albumin; Ca, serum calcium; P, serum phosphorus; Chole, cholesterol; BMI-z, body mass index z-score. The first column of each angle is the *r*-value and the second column is the *p*-value.

**Table 8:** Comparisons of characteristics of patients with iron deficiency in the CKD cohort.

Variable	Patients with Iron Deficiency (*n* = 107)	Patients without Iron Deficiency (*n* = 290)	*P* Value
**Age (years)**	107	290	0.002 ††
0–2 years	16 (15.0)	18 (6.2)	
2–6 years	25 (23.4)	42 (14.5)	
6–12 years	30 (28.0)	85 (29.3)	
12–17 years	36 (33.6)	145 (50.0)	
**Sex (male/female)**	68/39	205/85	0.173 ††
**Disease preceding CKD**	95	254	0.017 ††
Glomerulonephritis	12 (12.6)	62 (24.4)	
Non-glomerulonephritis	83 (87.3)	192 (75.6)	
**CKD stages**	107	290	0.057 ††
I	23 (21.5)	46 (15.9)	
II	28 (26.2)	75 (25.9)	
IIIa ^※^	26 (24.3)	46 (15.9)	
IIIb ^※^	15 (14.0)	50 (17.2)	
IV	14 (13.1)	57 (20.0)	
V	1 (1.0)	16 (5.5)	
**Weight (kg)**	105	285	0.213 ††
Low weight	33 (30.8)	76 (26.7)	
Normal	66 (61.7)	201 (70.5)	
Overweight	6 (5.6)	8 (2.8)	
**Height (cm)**	105	285	0.835 †
Short stature	25 (23.8)	61 (21.4)	
Normal	78 74.3)	219 (76.8)	
Tall stature	2 (1.9)	5 (1.8)	
**Hypertension**	91	251	
SBP hypertension	20 (22.2)	32 (12.7)	0.036 ††
DBP hypertension	16 (17.6)	60 (23.9)	0.214 ††
**Comorbidity**	107	290	0.009 ††
Present	71 (66.4)	150 (51.7)	
Absent	36 (33.6)	140 (48.3)	
**Iron supplementation treatment**	107	289	0.692 ††
Yes **	21 (19.6)	62 (21.5)	

Numerical values are reported as *n* (%); CKD, chronic kidney disease; *n*, number of patients; EPO, erythropoietin; SBP, systolic blood pressure; DBP, diastolic blood pressure; ※ CKD stage III is divided into subgroup stage IIIa and IIIb according to the eGFR (estimated glomerular filtration rate), stage IIIa is an eGFR between 45 and 59 and Stage 3b is an eGFR between 30 and 44; ** Iron agents: Any agent including intravenous or oral intake; † Statistically significant differences were demonstrated using Fisher’s exact test; †† Statistical significance was demonstrated using the Chi-square test.

**Table 9:** Associated risk factors for iron deficiency in patients with CKD who did not take iron supplements.

	Univariate Logistic Regression in Iron Deficiency Patients		Multivariable Logistic Regression in Iron Deficiency Patients	
	Unadjusted OR (95% CI)	*P*-value	Adjusted OR (95% CI)	*P* Value
**Age**	0.908 (0.865, 0.954)	0.0001	-	-
**Weight z-score**	1.041 (0.895, 1.212)	0.6002	-	-
**Height z-score**	1.002(0.847, 1.184)	0.9856		
**BMI z-score**	1.148 (0.939, 1.403)	0.1783	1.318 (1.038, 1.675)	0.0235
**Birth age**	0.783 (0.545, 1.125)	0.1858	-	-
**Glomerulonephritis**	0.569 (0.277, 1.168)	0.1243	-	-
**Hypertension**	1.327 (0.752, 2.34)	0.3292	-	-
**Serum albumin**	0.791 (0.543, 1.152)	0.2209	-	-
**eGFR**	1.004 (0.996, 1.012)	0.3357	-	-
**iPTH**	0.997 (0.993, 1.001)	0.107	0.993 (0.988, 0.998)	0.007
**Serum Ca**	1.008 (0.804, 1.264)	0.9467	-	-
**Serum P**	1.09 (0.797, 1.493)	0.589	-	-
**Hemoglobin**	0.843 (0.734, 0.967)	0.0145	0.693 (0.565, 0.85)	0.0004
**Ferritin**	0.980 (0.970, 0.989)	<0.0001	0.976 (0.965, 0.987)	<0.0001
**Transferrin saturation**	1.018 (0.990, 1.047)	0.2164	-	-
Erythropoietin stimulating use **	0.985 (0.255, 3.802)	0.9825	-	-

CKD, chronic kidney disease; OR, odds ratio; CI, confidence interval; BMI, body mass index; GFR, glomerular filtration rate; iPTH, ionized parathyroid hormone; Ca, serum calcium; P, serum phosphorus; ** Iron agents: any agents including intravenous or oral intake.

## Supplementary Materials

The supplementary materials are available online at http://www.mdpi.com/2077-0383/8/2/152/s1.
