# Supplementary material for: Anemia and Iron Deficiency in Children with Chronic Kidney Disease (CKD): Data from the Know-Ped CKD Study
Source: J Clin Med. 2019 Jan 29;8(2):152. doi: 10.3390/jcm8020152 (PMC6406575; doi:10.3390/jcm8020152)
Supplement: Supplementary file 1 [file jcm-08-00152-s001.pdf]

Supplementary Tables

Anemia and Iron Deficiency in Children with Chronic Kidney Disease: Data from the Know-Ped CKD Study

Keum Hwa Lee[1,2, *], Eujin Park[3, *], Hyun Jin Choi[4], Hee Gyung Kang[4], Il-Soo Ha[4], Hae Il Cheong[4], Young Seo Park[5], Heeyeon Cho[6], Kyoung Hee Han[7], Seong Heon Kim[8], Min Hyun Cho[9], Joo Hoon Lee[5] and Jae Il Shin[1,2,10]

1. Department of Pediatrics, Yonsei University College of Medicine, Seoul, Republic of Korea
2. Division of Pediatric Nephrology, Severance Children's Hospital, Seoul, Republic of Korea
3. Department of Pediatrics, Hallym University Kangnam Sacred Heart Hospital, Seoul, Republic of Korea
4. Department of Pediatrics, Seoul National University Children's Hospital & Seoul National University College of Medicine, Seoul, Republic of Korea
5. Department of Pediatrics, Asan Medical Center Children's Hospital, University of Ulsan College of Medicine, Seoul, Republic of Korea
6. Department of Pediatrics, Samsung Medical Center, Sungkyunkwan University School of Medicine, Seoul, Republic of Korea
6. Department of Pediatrics, Jeju National University School of Medicine, Jeju, Republic of Korea
7. Department of Pediatrics, Pusan National University Children's Hospital, Yangsan, Republic of Korea
8. Department of Pediatrics, Kyungpook National University, School of Medicine, Daegu, Republic of Korea
9. Department of Pediatrics, Asan Medical Center, University of Ulsan College of Medicine, Seoul, Republic of Korea
10. Institute of Kidney Disease Research, Yonsei University College of Medicine, Seoul, Republic of Korea

*These authors are contributed equally to this work.

Corresponding author: Joo Hoon Lee and Jae Il Shin, M.D., Ph.D.
Dr. Joo Hoon Lee
Department of Pediatrics, Asan Medical Center Children's Hospital, University of Ulsan College of Medicine, 88, Olympic-ro 43-Gil, Songpa-Gu, Seoul 138-736, Republic of Korea.
Dr. Jae Il Shin
50 Yonsei-ro, Seodaemun-gu, C.P.O. Box 8044, Department of Pediatrics, Yonsei University College of Medicine, Seoul 120-752, Republic of Korea

Supplementary Table S1. Distribution of causative disease of primary and secondary glomerulopathy preceding CKD

		N
**Primary glomerulopathy (N=61)**	FSGS	25
	IgA nephropathy	19
	Membranoproliferative GN type1	2
	Dense deposit disease	2
	Membranoproliferative GN type3	2
	Mesangial proliferative GN	3
	Minimal change nephrotic syndrome	7
	Other glomerulopathy	1
**Second glomerulopathy (N=60)**	Henoch Schoenlein purpura nephritis	5
	Lupus nephritis	1
	Hemolytic uremic syndrome	11
	Polyarteritis	1
	Alport syndrome	22
	Congenital NS of Finnish type	2
	AD-FSGS 1 due to ACTN4 mutation	2
	Denys-Drash syndrome	1
	Frasier syndrome	1
	Congenital NS of other/ unknown genetic cause	5
	Renal artery stenosis occlusion	6
	Cardiorenal syndrome	3

CKD, chronic kidney disease; FSGS, focal segmental glomerulosclerosis; IgA, immunoglobulin A; GN, glomerulonephritis; \
AD, autosomal dominant; ACTN4, gene name encoding actinin-4 protein; NS, nephrotic syndrome
